# A novel panel of biomarkers in distinction of small well-differentiated HCC from dysplastic nodules and outcome values

**DOI:** 10.1186/1471-2407-13-161

**Published:** 2013-03-27

**Authors:** Guang-Zhi Jin, Hui Dong, Wen-Long Yu, Yan Li, Xin-Yuan Lu, Hua Yu, Zhi-Hong Xian, Wei Dong, Yin-Kun Liu, Wen-Ming Cong, Meng-Chao Wu

**Affiliations:** 1 Department of Pathology, Eastern Hepatobiliary Surgery Hospital, Second Military Medical University, Shanghai, 200438, China; 2 Department II of billiary tract Surgery, Eastern Hepatobiliary Hospital, Second Military Medical University, Shanghai, 200438, China; 3 Liver Cancer Institute, Zhong Shan Hospital & Institutes of Biomedical Sciences, Fudan University, Shanghai, 200032, China; 4 Department of Surgery, Eastern Hepatobiliary Surgery Hospital, Second Military Medical University, Shanghai, 200438, China

**Keywords:** High grade dysplastic nodules, Well-differentiated hepatocellular carcinoma, Aminoacylase-1, Sequestosome-1, Glypican-3

## Abstract

**Background:**

Differential diagnosis of high-grade dysplastic nodules (HGDN) and well-differentiated hepatocellular carcinoma (WDHCC) represents a challenge to experienced hepatic clinicians, radiologists and hepatopathologists.

**Methods:**

The expression profiles of aminoacylase-1 (ACY1), sequestosome-1 (SQSTM1) and glypican-3 (GPC3) in low-grade dysplastic nodules (LGDN), HGDN and WDHCC were assessed by immunohistochemistry. The differential diagnostic performances of these three markers alone and in combination for HGDN and WDHCC were investigated by logistic regression models (HGDN = 21; WDHCC = 32) and validated in an independent test set (HGDN, n = 21; WDHCC n = 24). Postoperative overall survival and time to recurrence were evaluated by univariate and multivariate analyses in an independent set of 500 patients.

**Results:**

ACY1, SQSTM1 and GPC3 were differentially expressed in each group. For the differential diagnosis of WDHCC from HGDN, the sensitivity and specificity of the combination of ACY1 + SQSTM1 + GPC3 for detecting WDHCC were 93.8% and 95.2% respectively in the training set, which were higher than any of the three two-marker combinations. The validities of the four diagnostic models were further confirmed in an independent test set, and corresponding good sensitivity and specificity were observed. Interestingly, GPC3 expression in HCC tissues combined with serum α-fetoprotein (AFP) was found to be an independent predictor for overall survival and time to recurrence.

**Conclusions:**

ACY1 + SQSTM1 + GPC3 combination represents a potentially valuable biomarker for distinguishing between WDHCC and HGDN using immunohistochemistry. Meanwhile, low GPC3 staining combined with positive serum AFP may play a practical role in predicting poor postoperative outcome and high tumor recurrence risk.

## Background

Hepatocellular carcinoma (HCC) is one of the most prevalent human cancers worldwide, with 82% of cases occurring in developing countries, including 55% in China) [1]. HCC occurs mainly in patients with chronic liver diseases such as hepatitis B virus or hepatitis C virus infection-based liver cirrhosis. Dysplastic nodules (DN) are pre-cancerous lesions of HCC and high-grade DN (HGDN) has a high risk of malignant transformation [2-5]. However, detection of DN, especially HGDN, and its differentiation from small well-differentiated HCC (WDHCC) are sometimes very difficult on the basis of morphologic features alone. Although recent advances in imaging techniques have increased the frequency of detection of small lesions, issues such as the low specificity of their identification remain to be resolved [6,7].

It has been reported that HSP70, glypican-3 (GPC3), glutamine synthetase (GS), CD31, α-smooth muscle actin and CD34 may serve as biomarkers for the differential diagnosis of HCC or WDHCC and DN or HGDN [8-13]. However, the sensitivity of the individual markers for distinguishing between WDHCC and HGDN were only 78.1% for HSP70, 59.4% for GS, and 68.8% for GPC3, respectively [10], and CD34 immunoreactivity may be increased in HGDN [14], which may influence the accuracy of the pathological diagnosis and subsequent therapy. There is thus a need to develop new markers for the differential diagnosis of HGDN and WDHCC.

Using the iTRAQ-2DLC-ESI-MS/MS technique, we recently identified 147 proteins, including 52 that were up-regulated and 95 that were down-regulated in small HCC, and identified aminoacylase-1 (ACY1) and sequestosome-1 (SQSTM1) as candidate immunohistochemical markers for distinguishing between small HCCs (<3 cm) and DN [15]. To the best of our knowledge, the relationship between ACY1 and SQSTM1 expression in small HCC and postoperative prognosis has not yet been studied, and few studies, with only small sample sizes, have described the prognostic role of GPC3 in patients with HCC [16-19].

In the present study, we therefore analyzed the expression patterns of ACY1, SQSTM1, and GPC3 among low-grade DN (LGDN), HGDN, WDHCC and moderately differentiated HCC (MDHCC), and determined the accuracies of different panels of markers using ACY1, SQSTM1, and GPC3. In addition, we established four differential diagnostic models by logistic regression analyses to evaluate their diagnostic values for distinguishing small WDHCC from HGDN, and externally validated the results in an independent set of 45 samples. We also evaluated the prognostic values of ACY1, SQSTM1 and GPC3, and demonstrated that GPC3 combined with serum α-fetoprotein (AFP), and TNM stage were independent prognostic factors for overall survival (OS) and time to recurrence (TTR).

## Methods

### Patients and specimens

A total of 129 formalin-fixed paraffin-embedded (FFPE) tissues from liver nodules (diagnostic group; LGDN = 25, HGDN = 42, WDHCC = 56, MDHCC = 19) were randomly selected retrospectively from patients who underwent curative resection between 2005 and 2011 at the Eastern Hepatobiliary Surgery Hospital (EHBH), Second Military Medical University, Shanghai, China (diagnostic group in Additional file 1: Table S1). An additional cohort of 500 FFPE tissues was randomly selected retrospectively from HCC patients who underwent curative resection from January 1996 to September 2001 in the same hospital as a follow up group (prognostic group in Additional file 1: Table S1). Complete follow-up data were available for patients in the prognostic group. Patients were followed until October 2008, with a median follow-up of 33.0 months (range, 0.3–141.0 months). Computed tomography and/or magnetic resonance imaging and an elevated serum AFP level (>20 ng/ml) were used to verify tumor recurrence in suspected cases.

Hematoxylin and eosin (HE)-stained slides were made from each FFPE tissue sample and were reviewed by two experienced hepatopathologists (WM-Cong and H-Dong). Diagnoses of LGDN and HGDN were based on the criteria proposed by the International Consensus Group for Hepatocellular Neoplasia (ICGHN) and the World Health Organization (WHO) [20,21]. Briefly, hepatocytes in LGDN appear normal or show minimal nuclear atypia and a slightly increased nucleus to cytoplasmic (N:C) ratio, but mitotic figures are absent. HGDN is characterized by cytologic and/or structural atypia, but insufficient for a diagnosis of WDHCC. The cytologic atypia may be diffuse or focal and is characterized by nuclear hyperchromasia, nuclear contour irregularities, cytoplasmic basophilia or clear cell changes, high N:C ratio, and occasional mitotic figures. Architecturally, the cell plates are thickened up to three cells, with occasional foci of pseudoglandular formation. All WDHCC and MDHCC in the diagnostic group were <3 cm in diameter. WDHCC was diagnosed mainly based on the following major histologic features proposed by ICGHN and WHO: (1) increased cell density, more than twice that of the surrounding liver, with increased N:C ratio; (2) irregular thin-trabecular pattern of growth; (3) pseudoglandular structures; (4) fatty change; (5) unpaired arteries; (6) intratumoral portal tracts; and (7) stromal invasion [20,21]. Tumor stage was defined according to the 2002 American Joint Committee on Cancer/International Union Against Cancer tumor node metastasis (TNM) classification system [22].

A total of 642 specimens obtained from 632 patients were therefore used in the present study. Among these, 142 specimens were included in the diagnostic group and 500 in the prognostic group. The baseline characteristics of the patients are summarized in Additional file 1: Table S1. Approval from the Ethics Committee of EHBH and written informed consent from each patient were obtained prior to the use of these clinical materials for investigation.

### Tissue microarrays, immunohistochemistry and scoring

Tissue microarrays were constructed as reported previously [23], using 597 samples selected randomly from 642 specimens. The remaining 45 specimens, including 21 HGDN and 24 WDHCC specimens were used for the diagnostic validation set. HE-stained slides from all patients were reviewed and identified by two experienced pathologists (WM-Cong. and H-Dong) and the representative two cores were pre-marked in the paraffin blocks. Tissue cylinders with a diameter of 2 mm were punched from the marked areas of each block and incorporated into a recipient paraffin block. Sections 4-μm thick were placed on slides coated with 3-aminopropyltriethoxysilane. Paraffin sections were deparaffinized in xylene and rehydrated through decreasing concentrations of ethanol (100%, 95%, and 85%, 5 min each). Antigens were unmasked by microwave irradiation for 3 min in pH 6.0 citric buffer and cooled at room temperature for 60 min. Endogenous peroxidase activity was blocked by incubation of the slides in 3% H_2_O_2_/phosphate-buffered saline, and nonspecific binding sites were blocked with goat serum. Primary antibodies were diluted as follows: mouse monoclonal antibody against ACY1 (ab54960; Abcam, Hong Kong, China; 1:250 dilution, cytoplasmic staining), mouse polyclonal antibody against SQSTM1 (P0067; Sigma-Aldrich, St. Louis, MO, USA); 1:1000 dilution, cytoplasmic staining), mouse monoclonal antibody against GPC3 (Clone 1G12; BioMosaics, USA; 1:200 dilution, cytoplasmic staining).

An EnVision Detection kit (GK500705: Gene Tech, Shanghai, China) was used to visualize tissue antigens. Tissue sections were counterstained with hematoxylin for 5 min. Negative control slides omitting the primary antibodies were created for all assays. The integrated optical density (IOD) as the positive-staining density was measured as reported previously [24]. The image system comprised a Leica CCD camera DFC420 connected to a Leica DM IRE2 microscope (Leica Microsystems Imaging Solutions Ltd, Cambridge, United Kingdom). Photographs of representative fields were captured under high-power magnification (×200) using Leica QWin Plus v3 software. The IODs of all the negative- and positive-stained regions in each photograph were counted and measured using Image-Pro Plus v6.0 software (Media Cybernetics Inc, Bethesda, MD, USA). In addition, cases were semiquantitatively evaluated by two pathologists (WM-Cong. and H-Dong) who were blinded to the clinicopathological data. The intensity of immunostaining was scored on the basis of the percentage of positive tumor cells: 0 (−) (0–15%), 1 (+) (16–25%), 2 (++) (26–50%), and 3 (+++) (>51%) for ACY1 and SQSTM1 and 0 (−) (0–5%), 1 (+) (6–10%), 2 (++) (11–50%), and 3 (+++) (>51%) for GPC3.

### Construction of diagnostic models and validation of diagnostic efficiency

HGDN (n = 21) and WDHCC (n = 32) scores from immunohistochemistry were used to construct diagnostic models (training data set from tissue microarray). The scores (0, 1, 2, 3) for ACY1, SQSTM1, and GPC3 were subjected to logistic regression to generate differential diagnostic models for the detection of WDHCC. The output was the diagnostic score in the range of 0–1. During model construction, the diagnostic score for an HGDN lesion was defined as ‘0’, while that for a WDHCC lesion was defined as ‘1’. The predictive probability of this model was applied to the same data set (HGDN = 21, WDHCC = 32), and receiver operator characteristic curve (ROC) analysis was performed.

The differential diagnostic models were then applied to classify the HGDN and WDHCC cases in the independent validation set (HGDN = 21, WDHCC = 24). The diagnostic scores, which were computed from the model using the immunostaining scores for ACY1, SQSTM1, and GPC3 in individual cases, were used as an index for classifying the WDHCC and HGDN.

### Statistical analyses

Statistical analyses were carried out using SPSS 13.0 software (SPSS, Chicago, IL, USA). The relationships between the expression of biomarkers and hepatocellular tumors (LGDN, HGDN, WDHCC, and MDHCC) were analyzed by calculating Spearman’s correlation coefficient (r). Quantitative variables were analyzed using Student’s *t*-test or the Mann–Whitney test. Experimental data were presented as the mean of each condition ± S.D. or S.E.M, and values of p < 0.05 were considered statistically significant. ROC curves were used to determine the sensitivity, specificity, and corresponding cut-off value for each marker or panel of markers [25].

For survival analyses, ACY1, SQSTM1, and GPC3 expression levels were divided into low and high levels as follows: ACY1: low (−), high (+, ++); SQSTM1: low (−, +), high (++, +++); GPC3: low (−, +), high (++, +++). Univariate analysis was performed using the Kaplan-Meier method (log-rank test). Multivariate analysis was performed using Cox’s multivariate proportional hazards regression model in a stepwise manner (forward, conditional likelihood ratio).

## Results

### Features of expression profiles

**Figure 1:**
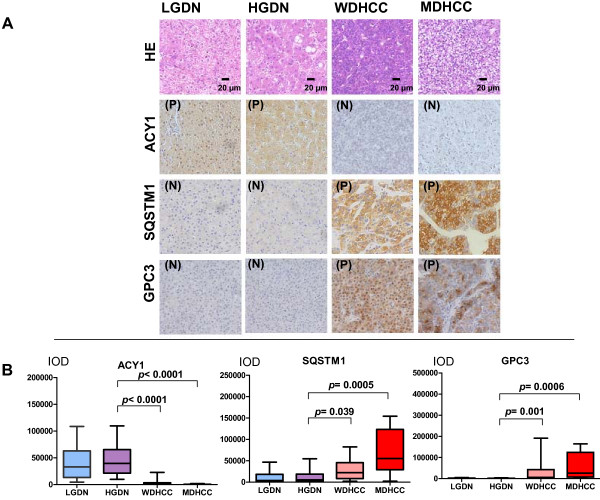
**Representative HE-stained sections and immunohistochemical staining for ACY1, SQSTM1, and GPC3 (×200).** (**A**) Typical HE-stained sections and immunostaining for ACY1, SQSTM1, and GPC3 are shown for LGDN, HGDN, WDHCC, and MDHCC. P, positive immunostaining; N, negative immunostaining. (**B**) Immunohistochemical expression of ACY1, SQSTM1, and GPC3 in LGDN, HGDN, WDHCC, and MDHCC. A box and whisker plot (whiskers: 10–90%) of IOD for each marker was obtained from the tissue microarrays. Mann–Whitney tests showed a significant difference between WDHCC (32 lesions) and MDHCC (19 lesions) compared with HGDN (21 lesions).

The expression levels of ACY1 in WDHCC and MDHCC were lower than in LGDN and HGDN. In contrast, the expression levels of SQSTM1 and GPC3 were higher in WDHCC and MDHCC than in LGDN and HGDN (Figure 1A). As shown in Figure 1B, the expression levels (based on IOD) of ACY1 in WDHCC and MDHCC were significantly lower than in HGDN, and SQSTM1 and GPC3 were significantly higher in WDHCC and MDHCC than in HGDN. The immunoreactivity score distribution of ACY1 decreased significantly in line with the stepwise progression of hepatocarcinogenesis (from LGDN to MDHCC) (Spearman’s r = −0.639, p < 0.0001), whereas SQSTM1 and GPC3 increased significantly in line with the same progression (Spearman’s r = 0.644 for SQSTM1; Spearman’s r = 0.616 for ACY1, p < 0.0001 for both). The proportion of positive immunoreactivity also showed stepwise changes; for instance, negative immunoreactivity for SQSTM1 was demonstrated in 84.0% of LGDN, 81.0% of HGDN, 15.6% of WDHCC, and 15.8% of MDHCC (Table 1).

**Table 1:** Immunoreaction score distribution of ACY1, SQSTM1, and GPC3 according to histologic grade in LGDN, HGDN, WDHCC, and MDHCC

			**ACY1**	***%***	**SQSTM1**	***%***	**GPC3**	***%***
25	**LGDN**	-	**3**	12.0	21	84.0	25	100.0
	**(n = 25)**	**+**	**16**	*64.0*	**2**	*8.0*	**0**	*0.0*
		++	**6**	*24.0*	**2**	*8.0*	**0**	*0.0*
		**+++**	**0**	*0.0*	**0**	0.0	**0**	*0.0*
**21**	**HGDN**	-	**1**	*4.8*	**17**	*81.0*	**20**	*95.2*
	**(n = 21)**	**+**	**14**	*66.7*	**2**	*9.5*	**1**	*4.8*
		++	**6**	*28.6*	**2**	*9.5*	**0**	*0.0*
		**+++**	**0**	*0.0*	**0**	*0.0*	**0**	*0.0*
**32**	**WDHCC**	-	**24**	*75.0*	**5**	*15.6*	**12**	*37.5*
	**(n = 32)**	**+**	**6**	*18.8*	**12**	*37.5*	**11**	*34.4*
		++	**2**	*6.3*	**11**	*34.4*	**5**	*15.6*
		**+++**	**0**	*0.0*	**4**	*12.5*	**4**	*12.5*
**19**	**MDHCC**	-	**17**	*89.5*	**3**	*15.8*	**6**	*31.6*
	**(n = 19)**	**+**	**2**	*10.5*	**3**	*15.8*	**3**	*15.8*
		++	**0**	*0.0*	**3**	*15.8*	**7**	*36.8*
		**+++**	**0**	*0.0*	**10**	*52.6*	**3**	*15.8*
	**r**		**−0.639**		**0.644**		**0.616**	
	***p***		**< 0.0001**		**< 0.0001**		**< 0.0001**	

NOTE. LGDN, low grade dysplastic nodule; HGDN, high grade dysplastic nodule; WDHCC, welldifferentiated hepatocellular carcinoma; MDHCC, moderately differentiated hepatocellular carcinoma; r, correlation coefficient; *p,* spearman correlation (from LGDN to MDHCC).

### Significance of diagnostic models

**Figure 2:**
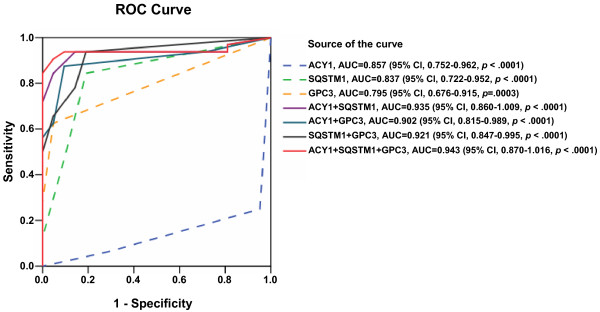
**ROC curve analysis of individual markers and combinations of ACY1, SQSTM1, and GPC3 for discriminating between WDHCC and HGDN lesions.** AUCs were 0.857 for ACY1, 0.837 for SQSTM1, 0.795 for GPC3, 0.935 for ACY1 + SQSTM1, 0.902 for ACY1 + GPC3, 0.921 for SQSTM1 + GPC3, 0.943 for ACY1 + SQSTM1 + GPC3.

To enhance the diagnostic efficiency, logistic regression analyses were used to construct four diagnostic models using the immunohistochemistry scores (HGDN = 21, WDHCC = 32), and the best cut-off values were determined by ROC curves. The areas under the curve (AUC) were 0.857 (95% CI, 0.752–0.962, p < 0.0001) for ACY1, 0.837 (95% CI, 0.722–0.952, p < 0.0001) for SQSTM1, and 0.795 (95% CI, 0.676–0.915, p = 0.0003) for GPC3 (Figure 2). However, the AUCs were 0.935 (95% CI, 0.860–1.009, p < 0.0001, cut-off value = 0.6585) for ACY1 + SQSTM1 combination, 0.902 (95% CI, 0.815-0.989, p < 0.0001, cut-off value = 0.5335) for ACY1 + GPC3 combination, 0.921 (95% CI, 0.847–0.995, p < 0.0001, cut-off value = 0.3226) for SQSTM1 + GPC3 combination, and 0.943 (95% CI, 0.870–1.016, p < 0.0001, cut-off value = 0.6366) for ACY1+ SQSTM1 + GPC3 combination, suggesting that the AUCs for marker combinations were much higher than those for any individual marker. ACY1 + SQSTM1 + GPC3 combination was better than any two-marker combination. The resulting diagnostic models are summarized in Additional file 1: Table S2.

### Values of marker combinations

The sensitivity, specificity, and positive and negative predictive values of the individual markers and four models for WDHCC detection are summarized in Table 2. Good sensitivity (84.4%) coupled with good specificity (81.0%) for WDHCC detection was seen for SQSTM1 alone. The sensitivities and specificities of ACY1 (negative) and GPC3 (positive) for the detection of WHHCC were 75.0% and 95.2%, and 62.5% and 95.2%, respectively. However, the sensitivities and specificities for discriminating between WDHCC and HGDN were 84.4% and 95.2% for ACY1 + SQSTM1 combination, 87.5% and 61.9% for ACY1 + GPC3 combination, 93.8% and 81.0% for SQSTM1 + GPC3 combination, and 93.8% and 95.2% for ACY1+ SQSTM1 + GPC3 combination. Notably, the sensitivity and specificity for discriminating between WDHCC and HGDN were significantly improved by combining ACY1 + SQSTM1 + GPC3.

**Table 2:** Sensitivity, specificity, positive and negative predictive values for WDHCC detection using individual markers and marker combinations

	**WDHCC (n = 32)**	**HGDN (n = 21)**	**Sen**	**Spe**	**PPV**	**NPV**
ACY1 negative	24	1	75.0%	95.2%	96.0%	71.4%
SQSTM1 positive	27	4	84.4%	81.0%	87.1%	77.3%
GPC3 positive	20	1	62.5%	95.2%	95.2%	95.2%
Predicted by ACY1 + SQSTM1	27	20	84.4%	95.2%	96.4%	80.0%
Predicted by ACY1 + GPC3	28	13	87.5%	61.9%	77.8%	76.5%
Predicted by SQSTM1 + GPC3	30	17	93.8%	81.0%	88.2%	89.5%
Predicted by ACY1 + SQSTM1 + GPC3	30	20	93.8%	95.2%	96.8%	90.9%

NOTE. LGDN, low grade dysplastic nodule; HGDN, high grade dysplastic nodule; WDHCC, welldifferentiated hepatocellular carcinoma; MDHCC, moderately differentiated hepatocellular carcinoma; Sen, sensitivity; Spe, specification; PPV, positive predictive value; NPV, negative predictive value.

### Model evaluation

**Figure 3:**
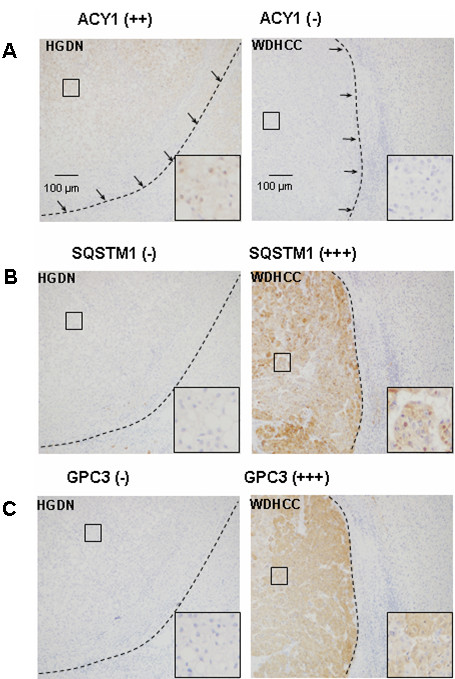
**Expression patterns of three biomarkers in large sections of HGDN and WDHCC.** Expression patterns of ACY1 (**A**), SQSTM1 (**B**), and GPC3 (**C**) examined by immunohistochemistry (×200) in HGDN and WDHCC validation set. Dotted line indicates the boundary between the tumor (HGDN and WDHCC) and the non-tumor tissues. Insert shows high magnification image.

The four models were evaluated by applying them to the independent sample set. We tested the expression profiles of the three markers (ACY1, SQSTM1, and GPC3) in a validation set of HGDN (n = 21) and WDHCC (n = 24). Typical immunostaining of serial large sections of HGDN and WDHCC is shown in Figure 3. As in tissue microarray analyses, ACY1 was significantly down-regulated in HCC compared with HGDN, while SQSTM1 and GPC3 were significantly up-regulated in HCC compared with HGDN. The immunostaining scores for ACY1, SQSTM1, and GPC3 in individual cases were used as indexes for classifying WDHCC (n = 24) and HGDN (n = 21). Finally, 79.2% of WDHCCs (19/24) and 57.1% of HGDNs (12/21) were correctly classified by the ACY1 + SQSTM1, 83.3% of WDHCCs (20/24) and 57.1% of HGDNs (12/21) by ACY1 + GPC3, 83.3% of WDHCCs (20/24) and 90.5% of HGDNs (19/21) by SQSTM1 + GPC3, and 79.2% of WDHCCs (19/24) and 95.2% of HGDNs (20/21) by ACY1 + SQSTM1 + GPC3. Notably, the SQSTM1 + GPC3 and ACY1 + SQSTM1 + GPC3 combinations demonstrated high sensitivity and good specificity for discriminating between WDHCC and HGDN (Additional file 1: Table S3).

### Prognostic significance

**Figure 4:**
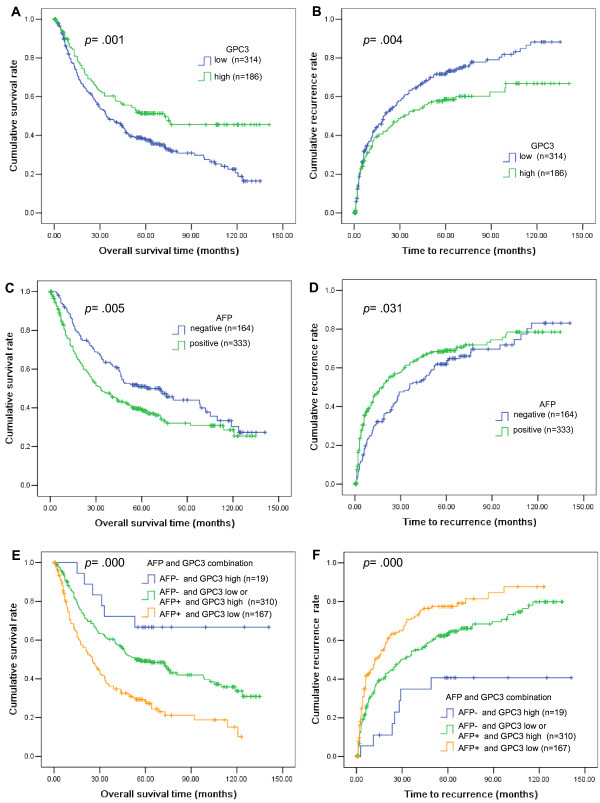
**Kaplan-Meier curves of survival differences among HCC patients.** OS and TTR for GPC3 expression in HCC tissue (**A** and **B**) and serum AFP levels (**C** and **D**) were significantly different (log-rank test), while serum AFP combined with GPC3 (**E** and **F**) were highly significantly different (log-rank test).

At the time of the last follow-up, 312 of 500 patients had tumor recurrence and 279 patients had died, including 34 patients with no record of tumor recurrence. Univariate analysis (Kaplan-Meier analysis) showed that the median OS time for patients with HCC expressing low levels of GPC3 was 34.3 (95% CI, 25.9–42.7) months, compared with 72.3 (95% CI, 48.3–99.4) months for patients with HCC expressing high levels of GPC3 (p = 0.001; log-rank test; Figure 4A). The median TTR for patients with HCC expressing low levels of GPC3 was 19.2 (95% CI, 13.1–25.3) months, compared with 32 (95% CI, 16.9–47.1) months for patients with HCC expressing high levels of GPC3 (p = 0.004; log-rank test; Figure 4B). However, ACY1, and SQSTM1 had no prognostic significance for OS and TTR (Additional file 1: Figure S1A–D). Furthermore, serum AFP (Figure 4C), TNM stage, tumor differentiation, and vascular invasion (Additional file 1: Figure S1E, G, I) were also significantly associated with OS, and serum AFP (Figure 4D), TNM stage, vascular invasion (Additional file 1: Figure S1F, J) were significantly associated with TTR. The median OS for patients who were negative for serum AFP was 72.6 (95% CI, 48.9–96.3) months, compared with 33.3 (95% CI, 24.0–42.6) months for serum AFP-positive patients (p = 0.005; log-rank test; Figure 4C). The median OS times for TNM stage, tumor differentiation, and vascular invasion were: TNM state, I vs II vs III–IV = 72.6 vs 44 vs 13.3 months; tumor differentiation, well vs moderate vs poor = 80.8 vs 40.4 vs 12.8 months; and vascular invasion, no vs yes = 72.3 vs 32.3 months. In addition, Kaplan-Meier analysis showed that sex, age, hepatitis B surface antigen (HBsAg), cirrhosis, and Child-Pugh class had no prognostic significance for OS and TTR. Tumor differentiation was not associated TTR.

Interestingly, when GPC3 staining and serum AFP were considered together, the OS and TTR rates were significantly better in AFP-negative/GPC3-high patients compared with AFP-positive/GPC3-low patients, while AFP-negative/GPC3-low and AFP-positive/GPC3-high patients showed intermediate OS and TTR rates. Further, multivariate Cox regression analysis indicated that, as for TNM stage, GPC3 staining combined with serum AFP was an independent prognostic factor for postoperative outcome and tumor recurrence in HCC patients (Table 3).

**Table 3:** Univariate and multivariate analyses of factors associated with OS and TTR

			**OS**				**TTR**	
			**Multivariate**				**Multivariate**	
**Factors**	**Univariate *p***	**HR**	**95% Cl**	***p***	**Univariate *p***	**HR**	**95% Cl**	***p***
Sex: Male *vs* Female	0.870			NA	0.547			NA
Age: < 50 *vs* >50	0.241			NA	0.131			NA
HBsAg: positive *vs* negative	0.166			NA	0.178			NA
Cirrhosis: yes *vs* no	0.077			NA	0.135			NA
serum AFP (ng/ml): ≤ 20 *vs* > 20	**0.005**			NA	**0.031**			NA
Child-pugh: A *vs* B *vs* C	0.284			NA	0.225			NA
TNM: I *vs* II *vs* III-IV	**0.000**	1.534	1.262-1.864	**0.000**	**0.000**	1.496	1.243-1.801	**0.000**
tumor differentiation:								
well *vs* moderate *vs* Poor	**0.025**			NS	0.236			NA
vascular invasion: yes *vs* no	**0.002**			NS	**0.013**			NA
ACY1: low *vs* high	0.930			NA	0.687			NA
SQSTM1: low *vs* high	0.438			NA	0.932			NA
GPC3: low *vs* high	**0.001**			NS	**0.004**			NS
AFP and GPC3 combination								
A-/G high *vs* A-/G low or A+/ G high vs A+/G low	**0.000**	1.811	1.439-2.279	**0.000**	**0.000**	1.530	1.233-1.898	**0.000**

NOTE: Univariate analysis was calculated by the Kaplan–Meier method (the log-rank test). Multivariate analysis was done using the Cox multivariate proportional hazard regression model with stepwise manner (forward, likelihood ratio). AFP, α-fetoprotein; A-, AFP negative; A+, AFP positive; G high, GPC3 high; G low, GPC3 low; TTR, time to recurrence; OS, overall survival; NS, not significant; NA, not adopted; HR, hazard ratio; Cl, confidential interval.

## Discussion

Differentiating between HGDN and WDHCC represents a challenge even to experienced hepatic clinicians, radiologists and hepatopathologists, and the pathological differentiation of pre-neoplastic lesions, particularly HGDN and small WDHCC, is always questionable [20,21,26,27]. Although several immunohistochemical markers such as GPC3, HSP70, GS, and EZH2 have been reported to play roles in the diagnosis of HCC, some limitations remain [10,13,28]; e.g., the sensitivity and specificity of GPC3 for the diagnosis of small HCC were 77% and 96% respectively in resected cases [29], and 61.4% and 92% respectively in needle biopsies [13]. Based on our experience in EHBH, the immunohistochemical sensitivity of GPC3 in 3,232 cases of HCC (from August 2010 to July 2011) was only 63.1%, while those of HSP70 and GS were not as high as expected (data not shown). Such limitations may result in confusion between small WDHCC and HGDN.

In the present study, we used ACY1 and SQSTM1, which were initially identified by screening in our laboratory [15], and a ‘star molecule’ GPC3 to establish diagnostic panels to differentiate between HGDN and WDHCC using logistic regression analyses. The models were then further validated in an independent set of WDHCC and HGDN samples. ACY1, SQSTM1, and GPC3 expression differed significantly between WDHCC and HGDN (Additional file 1: Table S4). In addition, there were no differences in expression levels of ACY1, SQSTM1 or GPC3 in HCCs <2 cm or 2–3 cm in diameter (Additional file 1: Figure S2).

Moreover, the sensitivity and specificity of ACY1 + SQSTM1 + GPC3 were higher than those of any single marker or any two-marker combination, with a sensitivity and specificity of 93.8% and 95.2%, respectively, for this new diagnostic model of ACY1 + SQSTM1 + GPC3 combination, constructed by logistic regression. The immunostaining scores for ACY1, SQSTM1, and GPC3 can be input into Model 4 during routine daily practice. The model can be easily set up and processed using a workstation. An output value ≤0.6366 is considered highly predictive of HGDN, while an output >0.6366 predicts WDHCC. This three-marker combination (−/+++/+++) demonstrated the highest sensitivity and specificity in terms of diagnostic value for diagnosing HCC, especially early highly-differentiated HCC.

Tommaso et al. recently observed that the use of an additional marker (clathrin heavy chain) improved the performance (sensitivity) of the immunomarker panel GPC3 + HSP70 + GS [30]. We aim to investigate the use of additional markers, including those mentioned above, together with our previous proteomics results, to further improve the sensitivity and specificity of the marker panels.

We demonstrated that ACY1 was expressed at low levels in WDHCC, while SQSTM1 was expressed at high levels in WDHCC tissues, compared with LGDN and HGDN. ACY1 is a cytosolic, homodimeric, zinc-binding enzyme that catalyzes the hydrolysis of acylated L-amino acids to L-amino acids and acyl groups [31]. SQSTM1 is an adapter protein that binds ubiquitin and may regulate signaling cascades through ubiquitination. It may regulate the activation of nuclear factor-κB by tumor necrosis factor-α, nerve growth factor and interleukin-1 [32-34]. The present study demonstrated a gradual decrease in ACY1 expression and a gradual increase in SQSTM1 and GPC3 expression from LGDN to MDHCC, which were confirmed by Spearman correlations and were in accordance with the stepwise progression of hepatocarcinogenesis. Although ACY1 and SQSTM1 showed no prognostic values in this present study, they presented significant diagnostic values and raised the sensitivity of GPC3 for the detection of WDHCC.

GPC3 is a member of the glypican family of glycosyl-phosphatidylinositol-anchored cell surface heparan sulfate proteoglycans [35]. It is expressed in embryonic mesodermal tissues and plays an important role in embryonal growth [36,37]. In addition to HCC, GPC3 displays loss-of-function mutations in Simpson-Golabi-Behmel syndrome [36,37], and changes in GPC3 expression levels have been detected in lung squamous cell carcinomas [38]. In the present study, TNM stage and serum AFP were independent prognostic factors for OS and TTR, in agreement with previous reports [24,39,40]. Kaplan-Meier and multivariate survival analyses revealed that lower GPC3 expression was significantly linked to both poor OS and increased risk of recurrence after surgical resection in HCC patients. However, apart from studies on GPC3 staining in HCC tissues, few studies have reported any association between high GPC3 expression and poor outcome in HCC patients [16-19]. This discrepancy might be partly related to the following factors. The above studies were based on relatively small sample sizes (n = 61, 86, 107 and 185, respectively), and the use of different GPC3 scoring systems may lead to contradictory results for predicting long-term prognoses [18]. There may also have been differences between studies in terms of factors such as antibody sources and maximum follow-up time (Additional file 1: Table S5). In addition, age, HBsAg, serum AFP, TNM, and tumor differentiation differed significantly between GPC3-low and GPC3-high patients (Additional file 1: Table S6), and these results were similar to those from previous reports [16-18]. To the best of our knowledge, the present study evaluated GPC3 prognostic values using the largest sample size (n = 500) with the longest follow-up time (up to 12 years).

To date, few and limited data have been reported regarding the use of both serological and immunohistochemical biomarkers to predict postoperative prognosis in patients with HCC. As shown by Kaplan-Meier analysis, although either serum AFP or GPC3 staining alone had prognostic values, OS and TTR were lower in patients with both positive serum AFP and low GPC3 expression. In addition, TNM staging and serum AFP combined with GPC3 staining were adopted from Cox multivariate regression analyses, indicating that TNM and serum AFP/GPC3 staining may be a promising prognostic parameter in HCC patients undergoing surgical resection.

## Conclusions

In conclusion, the present study constructed a molecular model using logistic regression analysis for distinguishing between WDHCC and HGDN. The combination of ACY1 + SQSTM1 + GPC3 showed higher sensitivity and specificity than other reported panels, and we suggest that this combination represents a valuable differential diagnostic model in hepatic immunopathology. In addition, serum AFP positivity and low GPC3 staining is associated with poor prognosis, and can be a useful predictor to evaluate postoperative prognoses in patients with HCC.

## Competing interests

All the authors disclose no competing interests.

## Authors’ contributions

Conception and design: GZJ, WMC, YKL, MCW. Acquisition of data: GZJ, HD, XYL. Analysis and interpretation of data: GZJ, WMC, HD, YKL. Drafting of the manuscript: GZJ, WMC, YKL. Statistical analysis: GZJ, XYL. Critical revision of the manuscript for important intellectual content: WMC, YKL, MCW. Technical, or material support: GZJ, WD, ZHX, HY, HD, YL. Study supervision: WMC, YKL. All authors read and approved the final manuscript.

## Funding

This study was supported by the National Natural Science Foundation of China, No. 81201937, 81072026 and 81221061, and the Key Project of Science and Technology Committee of Shanghai, No. 10411951000, and the Major State Basic Research Development Program of China (973 Program) (2011CB910604).

## Pre-publication history

The pre-publication history for this paper can be accessed here:

http://www.biomedcentral.com/1471-2407/13/161/prepub

## Supplementary Material

Additional file 1: Table S1 Clinico-pathological features of the present series. **Table S2.** Resulted diagnostic models. **Table S3.** Histological diagnosis and diagnostic model diagnoses of the 45 nodules. **Table S4.** Chi-Square analysis of factors associated with HGDN and WDHCC. **Table S5.** Comparison of parameters in GPC3 related OS analyses among several study. **Figure S1.** Kaplan–Meier curves of survival differences among HCC patients. ACY1. **Figure S2.** Immunohistochemical expression of ACY1 (A), SQSTM1 (B), and GPC3 (C) in HCC which were divided into ≤2cm and 2cm< and ≤3cm. Integrated Optical Density (IOD) for each marker were obtained from the tissue microarrays. Mann-Whitney Test showed that no significant difference between two groups **Table S6.** Relationship between glypican-3 expression and clinicopathologic-features of HCC patients in prognosis group. (PDF 343 kb) Click here for file
