# Supplementary material for: A novel panel of biomarkers in distinction of small well-differentiated HCC from dysplastic nodules and outcome values
Source: BMC Cancer. 2013 Mar 27;13:161. doi: 10.1186/1471-2407-13-161 (PMC3621586; doi:10.1186/1471-2407-13-161)
Supplement: Additional file 1: Table S1 — Clinico-pathological features of the present series. Table S2. Resulted diagnostic models. Table S3. Histological diagnosis and diagnostic model diagnoses of the 45 nodules. Table S4. Chi-Square analysis of factors associated with HGDN and WDHCC. Table S5. Comparison of parameters in GPC3 related OS analyses among several study. Figure S1. Kaplan–Meier curves of survival differences among HCC patients. ACY1. Figure S2. Immunohistochemical expression of ACY1 (A), SQSTM1 (B), and GPC3 (C) in HCC which were divided into ≤2cm and 2cm< and ≤3cm. Integrated Optical Density (IOD) for each marker were obtained from the tissue microarrays. Mann-Whitney Test showed that no significant difference between two groups Table S6. Relationship between glypican-3 expression and clinicopathologic-features of HCC patients in prognosis group. (PDF 343 kb) [file 1471-2407-13-161-S1.pdf]

Supplementary Data

Supplementary Table S1. Clinico-pathological features of the present series

	Diagnostic group		Prognostic group
Variable	DN	HCC	HCC
Patients	59	73	500
No. of lesions	67	75	500
Sex			
Male	49	62	430
Female	10	11	70
Age			
Mean	53.3	53.6	50.1
SD	9.6	10.3	10.9
HBsAg	54	62	403
HCV-Ab	5	2	-
Cirrhosis	48	54	395
serum AFP			
≤ 20 ng/ml	33	48	333
>20ng/ml	25	24	164
DN grade			
LGDN	25	-	-
HGDN	42	-	-
DN with HCC	43	-	-
Tumor size			
≤2 cm	46	29	31
2.1-3 cm	13	46	52
>3 cm	8	-	417
Child-pugh class			
A	51	66	464
B	5	1	36
C	1	-	-
TNM			
I	-	66	150
II	-	1	281
III-IV	-	0	69
tumor differentiation			
well	-	56	60
moderate	-	19	418
Poor	-		17
vascular invasion			
yes	-	-	324
no	-	-	176

NOTE. HBsAg, hepatitis B virus surface antigen; SD, standard deviation; TNM, UICC TNM classification (6th edition).

Supplementary Table S2. Resulted diagnostic models.

Model 1 (ACY1+SQSTM1): $$P=\frac{e^{(0.926\ -2.546\times ACY1\ +\ 2.028\times SQSTM1)}}{1+e^{(0.926\ -2.546\times ACY1\ +\ 2.028\times SQSTM1)}}$$

Model 2 (ACY1+GPC3): $$P=\frac{e^{(1.136\ -1.936\times ACY1\ +\ 2.2\times GPC3)}}{1+e^{(1.136\ -1.936\times ACY1\ +\ 2.2\times GPC3)}}$$

Model 3 (SQSTM1+GPC3): $$P=\frac{e^{(-1.701\ +2.137\times GPC3\ +\ 1.665\times SQSTM1)}}{1+e^{(-1.701\ +2.137\times GPC3\ +\ 1.665\times SQSTM1)}}$$

Model 4 (ACY1+SQSTM1+GPC3): $$P=\frac{e^{(-0.105\ -1.897\times ACY1\ +\ 1.797\times SQSTM1+\ 1.589\times GPC3)}}{1+e^{(-0.105\ -\ 1.897\times ACY1\ +\ 1.797\times SQSTM1+\ 1.589\times GPC3)}}$$

NOTE. e is the mathematical constant and base value of natural logarithms; The immunostaining scores of ACY1, SQSTM1, and GPC3 of individual cases, enter the equations as index for classifying the WDHCC and HGDN.

Supplementary Table S3. Histological diagnosis and diagnostic model diagnoses of the 45 nodules.

		IHC score			predicted by							
No	Histology	ACY1	SQSTM1	GPC3	value	ACY1+SQSTM1	value	ACY1+GPC3	value	SQSTM1+GPC3	value	ACY1+SQSTM1+GPC3
1	HGDN	0	0	0	0.716	WDHCC	0.757	WDHCC	0.154	HGDN	0.474	HGDN
2	HGDN	0	0	0	0.716	WDHCC	0.757	WDHCC	0.154	HGDN	0.474	HGDN
3	HGDN	1	0	0	0.165	HGDN	0.310	HGDN	0.154	HGDN	0.119	HGDN
4	HGDN	2	0	0	0.015	HGDN	0.061	HGDN	0.154	HGDN	0.020	HGDN
5	HGDN	0	0	0	0.716	WDHCC	0.757	WDHCC	0.154	HGDN	0.474	HGDN
6	HGDN	0	1	1	0.950	WDHCC	0.966	WDHCC	0.891	WDHCC	0.964	WDHCC
7	HGDN	0	0	0	0.716	WDHCC	0.757	WDHCC	0.154	HGDN	0.474	HGDN
8	HGDN	2	0	0	0.015	HGDN	0.061	HGDN	0.154	HGDN	0.020	HGDN
9	HGDN	0	0	0	0.716	WDHCC	0.757	WDHCC	0.154	HGDN	0.474	HGDN
10	HGDN	1	0	0	0.165	HGDN	0.310	HGDN	0.154	HGDN	0.119	HGDN
11	HGDN	2	0	0	0.015	HGDN	0.061	HGDN	0.154	HGDN	0.020	HGDN
12	HGDN	1	0	0	0.165	HGDN	0.310	HGDN	0.154	HGDN	0.119	HGDN
13	HGDN	0	0	0	0.716	WDHCC	0.757	WDHCC	0.154	HGDN	0.474	HGDN
14	HGDN	0	0	0	0.716	WDHCC	0.757	WDHCC	0.154	HGDN	0.474	HGDN
15	HGDN	2	1	0	0.105	HGDN	0.061	HGDN	0.491	WDHCC	0.109	HGDN
16	HGDN	1	0	0	0.165	HGDN	0.310	HGDN	0.154	HGDN	0.119	HGDN
17	HGDN	1	0	0	0.165	HGDN	0.310	HGDN	0.154	HGDN	0.119	HGDN
18	HGDN	1	0	0	0.165	HGDN	0.310	HGDN	0.154	HGDN	0.119	HGDN
19	HGDN	1	0	0	0.165	HGDN	0.310	HGDN	0.154	HGDN	0.119	HGDN
20	HGDN	0	0	0	0.716	WDHCC	0.757	WDHCC	0.154	HGDN	0.474	HGDN
21	HGDN	1	0	0	0.165	HGDN	0.310	HGDN	0.154	HGDN	0.119	HGDN
						12/21 (57.1%)		12/21 (57.1%)		19/21 (90.5%)		20/21 (95.2%)

		IHC score				predicted by						
No	Histology	ACY1	SQSTM1	GPC3	value	ACY1+SQSTM1	value	ACY1+GPC3	value	SQSTM1+GPC3	value	ACY1+SQSTM1+GPC3
1	WDHCC	1	0	3	0.165	HGDN	0.997	WDHCC	0.997	WDHCC	0.941	WDHCC
2	WDHCC	2	0	0	0.015	HGDN	0.061	HGDN	0.061	HGDN	0.020	HGDN
3	WDHCC	1	0	3	0.165	HGDN	0.997	WDHCC	0.997	WDHCC	0.941	WDHCC
4	WDHCC	1	0	0	0.165	HGDN	0.310	HGDN	0.310	HGDN	0.119	HGDN
5	WDHCC	0	0	0	0.716	WDHCC	0.757	WDHCC	0.757	WDHCC	0.474	HGDN
6	WDHCC	0	2	1	0.993	WDHCC	0.966	WDHCC	0.966	WDHCC	0.994	WDHCC
7	WDHCC	0	0	0	0.716	WDHCC	0.757	WDHCC	0.757	WDHCC	0.474	HGDN
8	WDHCC	0	2	2	0.993	WDHCC	0.996	WDHCC	0.996	WDHCC	0.999	WDHCC
9	WDHCC	0	1	0	0.950	WDHCC	0.757	WDHCC	0.757	WDHCC	0.844	WDHCC
10	WDHCC	0	1	0	0.950	WDHCC	0.757	WDHCC	0.757	WDHCC	0.844	WDHCC
11	WDHCC	0	2	0	0.993	WDHCC	0.757	WDHCC	0.757	WDHCC	0.970	WDHCC
12	WDHCC	0	1	1	0.950	WDHCC	0.966	WDHCC	0.966	WDHCC	0.964	WDHCC
13	WDHCC	0	3	1	0.999	WDHCC	0.966	WDHCC	0.966	WDHCC	0.999	WDHCC
14	WDHCC	0	2	0	0.993	WDHCC	0.757	WDHCC	0.757	WDHCC	0.970	WDHCC
15	WDHCC	0	1	0	0.950	WDHCC	0.757	WDHCC	0.757	WDHCC	0.844	WDHCC
16	WDHCC	0	2	1	0.993	WDHCC	0.966	WDHCC	0.966	WDHCC	0.994	WDHCC
17	WDHCC	0	2	0	0.993	WDHCC	0.757	WDHCC	0.757	WDHCC	0.970	WDHCC
18	WDHCC	1	1	0	0.601	HGDN	0.310	HGDN	0.310	HGDN	0.449	HGDN
19	WDHCC	0	2	2	0.993	WDHCC	0.996	WDHCC	0.996	WDHCC	0.999	WDHCC
20	WDHCC	0	3	1	0.999	WDHCC	0.966	WDHCC	0.966	WDHCC	0.999	WDHCC
21	WDHCC	0	3	3	0.999	WDHCC	1.000	WDHCC	1.000	WDHCC	1.000	WDHCC
22	WDHCC	0	3	3	0.999	WDHCC	1.000	WDHCC	1.000	WDHCC	1.000	WDHCC
23	WDHCC	1	3	1	0.989	WDHCC	0.802	WDHCC	0.802	WDHCC	0.993	WDHCC
24	WDHCC	1	2	0	0.920	WDHCC	0.310	HGDN	0.310	HGDN	0.831	WDHCC
						19/24 (79.2%)		20/24 (83.3%)		20/24 (83.3%)		19/24 (79.2%)

NOTE. HGDN, high grade dysplastic nodule; WDHCC, welldifferentiated hepatocellular carcinoma; MDHCC; IHC score, immunohistochemical score. Optimal cut-off value determined from ROC analysis were 0.6585 for ACY1+SQSTM1combination, 0.5334 for ACY1+GPC3 combination, 0.3226 for SQSTM1+GPC3 combination, 0.6366 for ACY1+SQSTM1+GPC3 combination.

Supplementary Table S4. Chi-Square analysis of factors associated with HGDN and WDHCC

**Variable**		HGDN n = 21	WDHCC n = 32	Chi-Square test	*p*
Age, mean years		57.0	54.4	-	0.356*
Sex					
	M	20	25	1.72	0.190‡
	F	1	7		
HBsAg					
	positive	19	26	0.07	0.787‡
	negative	2	5		
serum AFP					
	positive	9	17	0.47	0.493†
	negative	11	14		
ACY1					
	positive	20	8	25.10	**0.000†**
	negative	1	24		
SQSTM1					
	positive	4	27	22.29	**0.000†**
	negative	17	5		
GPC3					
	positive	1	20	17.67	**0.000†**
	negative	20	12		

NOTE. HBsAg, hepatitis B virus surface antigen; HCV-Ab, hepatitis C virus antibody; HGDN, high grade dysplastic nodule; WDHCC, well differentiated hepatocellular carcinoma; *Student's *t* test; †Pearson X^2 test; ‡Continuity Correction.

Supplementary Table S5. Comparison of parameters in GPC3 related OS analyses among several study

	GPC3 antibody	OS patients	HBsAg positive proportion	HCV positive proportion	Score system	GPC3 positive	Follow upTime	Prognostic Valus
Shirakawa et al	Biomosaics	107	29/107 (27.1%)	62/107 (57.9%)	negative (<10%); positive (>10%)	87/107 (81.3%)	7 years	Yes (more high more poor)
Yorita et al	GC33	185	60/194 (31%)	83/194 (43%)			9 years	
					PR score	165/194 (85%)		No
					A-Cm score	152/194 (78.3%)		Yes (more high more poor)
					P-Cm score	104/194 (53.6%)		Yes (more high more poor)
Yu et al	Sigma	86	100/100 (100%)	-	-3 to +3	-	10 years	Yes (more high more poor)
Ning et al	Santa Cruz (sc-65443)	61	53/61 (86.9%)	-	low and high	-	5 years	Yes (more high more poor)
Present study	Biomosaics (1G12)	500	403/500 (80.6%)	-	low (-,+) and high (++,+++)	313/500 (62.5%)	12 yers	Yes (more low more poor)

NOTE. OS, overall survival; PR score, apparently positive area rate (0= negative, >1% positive); A-Cm score, positive area + staining pattern on cell membrane; P-Cm score, positive area + staining pattern on cell membrane + staining intensity on cell membrane.

Positive area: <20 % as 0, ≥20% as 1

Staining pattern on cell membrane: no circumferential pattern as 0, circumferential pattern <20% of positive cells as 1, ≥20% of positive cells as 2

Staining intensity on cell membrane: negative of faint as 0, easily recongnizable with a 4× objective lens as 1.

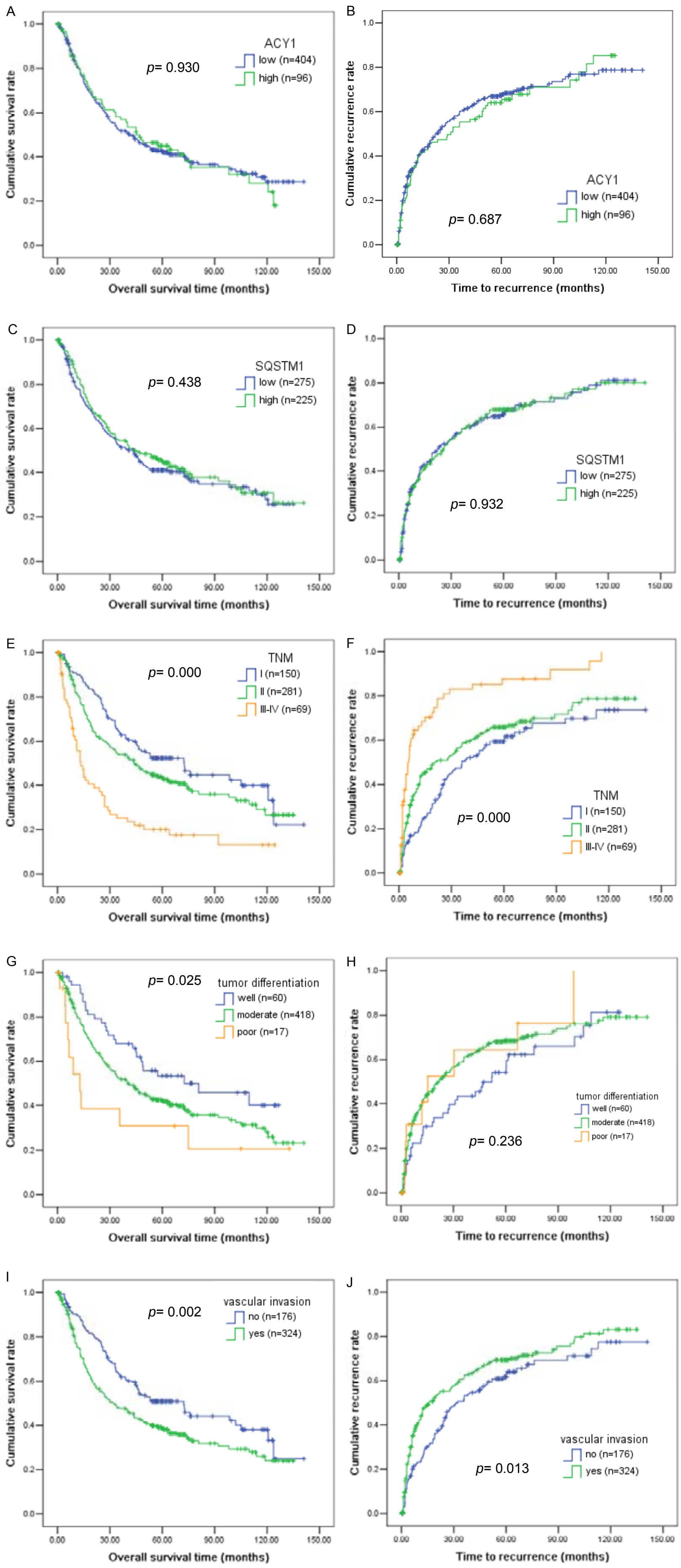

Supplementary Fig. S1. Kaplan–Meier curves of survival differences among HCC patients. ACY1

(A, B) and SQSTM1 (C, D) were not associated wit OS (overall survival time) and TTR (time to recurrence). TNM classification (E), tumor differentiation (G), and vascular invasion (I) were found to be significantly associated with OS (by the long-rank test). TNM classification (F), and vascular invasion (J) were found to be significantly associated with TTR (by the long-rank test), while, tumor differentiation (H) was not.

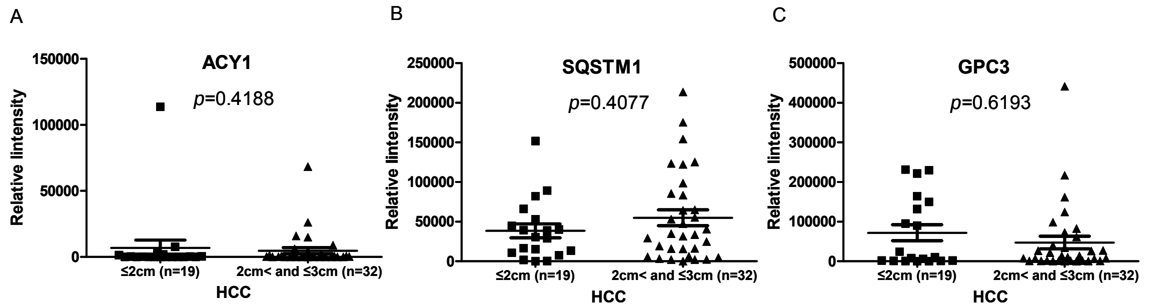

Supplementary Fig. S2. Immunohistochemical expression of ACY1 (A), SQSTM1 (B), and GPC3 (C) in HCC which were divided into ≤2cm and 2cm< and ≤3cm. Integrated Optical Density (IOD) for each marker were obtained from the tissue microarrays. Mann-Whitney Test showed that no significant difference between two groups.

Supplementary Table S6. Relationship between glypican-3 expression and clinicopathologic-features of HCC patients in prognosis group.

Variable	GPC3			
	All Cases	low expression	High expression	*p* Value
Sex				0 .063†
Male	430	277	153	
Female	70	37	33	
Age				**0.008†**
≤50	252	144	108	
＞50	248	170	78	
HBsAg				**0.012†**
negative	93	69	24	
positive	403	243	160	
Cirrhosis				0.810†
	105	67	38	
	395	247	148	
serum AFP				**0.000†**
≤20 ng/ml	164	145	19	
＞20ng/ml	333	167	166	
Tumor size				0.194†
≤2 cm	31	16	15	
2.1-3 cm	52	29	23	
＞3 cm	417	269	148	
Child-pugh class				0.565†
A	464	293	171	
B	36	21	15	
C	-		-	
TNM				0.163†
I	150	95	55	
II	281	169	112	
III-IV	69	50	19	
tumor differentiation				**0.000†**
well	60	53	7	
moderate	418	245	173	
Poor	17	12	5	
vascular invasion				0.919†
yes	324	204	120	
no	176	110	66	

NOTE. HBsAg, hepatitis B virus surface antigen; †Pearson X^2 test.
